# Analysis of Internal Training Load and Sports Injuries Incidence in Gymnasts of Different Exposure Levels

**DOI:** 10.3390/healthcare13131536

**Published:** 2025-06-27

**Authors:** Nicole Iasmim Minante da Silva, Zadriane Gasparetto, Sarita Mendonça Bacciotti, Rodolfo André Dellagrana, Gianfranco Sganzerla, Paula Felippe Martinez, Silvio Assis de Oliveira-Junior

**Affiliations:** 1 School of Physical Therapy, Federal University of Mato Grosso do Sul—UFMS, Campo Grande 79070-900, MS, Brazil; nicoleminante.ft@gmail.com (N.I.M.d.S.); paula.martinez@ufms.br (P.F.M.); 2 Graduate Program in Movement Sciences, Federal University of Mato Grosso do Sul—UFMS, Campo Grande 79070-900, MS, Brazil; zadriane@gmail.com (Z.G.); sarita.bacciotti@ufms.br (S.M.B.); radellagrana@uepg.br (R.A.D.); 3 Department of Physical Education, State University of Ponta Grossa, Ponta Grossa 84030-900, PR, Brazil; 4 Leisure and Sports Division, Pro-Rectorate of Community and Student Affairs, Federal University of Grande Dourados—UFGD, Dourados 79825-070, MS, Brazil; gianfrancosganzerla@ufgd.edu.br

**Keywords:** gymnastics, sports injuries, internal load, exercise training

## Abstract

**Background/Objectives:** Internal training load has been widely used to monitor training intensity and to prevent injuries in different sports. This study aimed to analyze the association between internal training parameters and sports injury incidence in gymnasts based on different training week exposure levels during a sequential sports season. **Methods:** The participants consisted of 27 gymnasts, aged 8 to 17 years old, recruited into two gymnastics training centers. The subjects were allocated into two groups: medium exposure (ME) and low exposure (LE) athletes. The monitoring period totaled 28 weeks. A survey was conducted to monitor sports injury incidence. The Perceived Effort Scale and the Total Quality of Recovery were applied to monitor training load and recovery, respectively. **Results:** A total of 28 injury cases were reported, with a higher incidence (24) in the ME group than in the LE group. Furthermore, the ME group demonstrated a significant increase in the average weekly load, as well as higher values of monotony and strain compared to the LE group (*p* < 0.001). The acute: chronic workload ratio (ACWR) was lower in the ME than in the LE group in all training periods. The total quality recovery (TQR) exhibited a peak during the competitive training period in the ME group, whereas strain showed a direct effect on this result. **Conclusions:** Increased training load values were linked to the incidence of musculoskeletal injuries in gymnasts submitted to different training week exposure levels. Likewise, the high values of internal training load were shown to be related to impaired recovery during a competitive period within a 28-week follow-up.

## 1. Introduction

Monitoring training load in sports is a fundamental background to identifying training effects and musculoskeletal injury prevention [1,2] for contributing to the onset of sports injuries during training or competitions. In this context, monitoring the external and internal load plays an important role for coaches and athletes. The external load is an objective variable presented by the athlete regardless of the internal characteristics that are usually measured through the different kinematic variables, such as speed, acceleration, and others [2,3]. Internal load, in turn, is linked to psychophysiological responses experienced by an athlete during the exercise, and it can be measured from several variables, including session rating of perceived exertion (RPE), total quality recovery (TQR) [3,4], and the acute: chronic workload ratio (ACWR) [5]. Despite being subjective variables, RPE and TQR are non-invasive and more sensitive than other objective measures [4]. The ACWR is currently used in sports sciences [5,6], and a greater risk of sustaining a time-loss injury has been associated with a higher ACWR score [7]. Thus, the ACWR can contribute to identifying periods of elevated injury risk based on excessive training loading [8].

However, the use of these tools to identify exercise training effects and the risk of sports injuries in youth athletes must be further studied. In particular, artistic gymnastics (AG) athletes take up sport at an early age, generally around six years old [9,10]. According to the Federation Internationale de Gymnastique (FIG), gymnastics athletes are allowed to participate in Olympic Games from 16 years old [11]. Aiming to reach a high-performance level, it is common to submit these athletes to 20–30 h weeks of exposure to AG training overload [10]. Hence, AG is a competitive sport, and the high risk of musculoskeletal injuries worries coaches, parents, and health professionals, as they can disrupt the development and maturation processes [12,13]. Recent studies have reported that women artistic gymnasts present fluctuations in external and internal training load variables over a season [14,15], which could be associated with multiple likelihood values for sports injury onset.

According to Freitas et al. [14], training load management must consider the specificity of demands in terms of training load; in addition, load monitoring, as well as ACWR, could measure exercise training effects. However, although some studies have documented internal load and training recovery in AG [1,16,17], their potential associations with sports injury incidence in gymnasts with different levels of exposure is yet to be determined.

This study evaluated internal training load and ACWR variables, as well as sports injury incidence in gymnasts with different levels of training exposure during a sequential sports season. Likewise, a second objective was to investigate potential adaptive changes in internal training load, as well as recovery quality scores and incidence of musculoskel-etal sports injuries in gymnasts with different training exposure over a 28-week training period. As an initial hypothesis, increased training exposure, as well as workload, would be more directly related to a significantly increased incidence of musculoskeletal injuries in these athletes compared to gymnasts with low exposure to exercise training.

## 2. Materials and Methods

### 2.1. Participants

A minimum of 24 participants were calculated for the study design with two independent groups and four comparative moments (Two-Way RM ANOVA). Other conditions included an alpha error of 0.05, power of 80%, as well as the ability to detect small differences (small effect size; d = 0.25) [18] between the groups, moments, and possible interaction. Sample size was calculated on the statistical program G*Power (version 3.1.9.7; Universität Kiel, Kiel, Germany).

Participants totalized 27 gymnasts (eight male and 19 female subjects) from two Brazilian young artistic gymnastics groups, located in the city of Campo Grande, state of Mato Grosso do Sul, Brazil. A convenience sample of subjects of both sexes, constituted by young gymnasts at the regional and national competitive levels (age: 10.3 ± 1.9 years; height: 1.4 ± 0.1 m; body weight: 36.4 ± 9.0 kg) participated in the study. Based on weekly training volume [19], participants were divided into Medium Exposure (ME; n = 16, 9–16 h/week) and Low Exposure (LE; n = 11, ≤6 h/week). All participants were in good health at the beginning of the study and were familiarized with the monitoring tools and provided written informed consent. This study was approved by the Ethics Committee in Research with Humans of the Federal University of Mato Grosso do Sul (CAAE 33562220.6.0000.0021; protocol 5013655, 2 October 2021).

### 2.2. Anthropometric Characteristics

For the anthropometric characterization, body weight was measured using a mechanical scale (Welmy R-110, Santa Bárbara D’oeste, SP, Brazil). Meanwhile, height was measured using a portable stadiometer (Sanny^®^, São Bernardo do Campo, SP, Brazil) with participants in an anatomical reference position and the head aligned in the Frankfurt plane [9]. Body mass index (BMI): BMI = mass (kg)/[height (m)^2^] [20] was calculated based on body weight and height. Additionally, triceps and subscapular skinfolds were measured to estimate body composition, while body adiposity (%F) was determined following Slaughter et al. [21].

### 2.3. Design and Training Periodization

A prospective study was performed during a macrocycle AG training with a 28-week duration, and participants were monitored across 195 training sessions and a competition session during a semester (between June to December 2022). Both training periodization schedules were based on a model proposed by Matveyev [22], typically moving from general training toward specific, pre-competitive, and competitive periods.

As to the ME group, the training week consisted of 3–6 sessions of three hours (Monday to Saturday; Rest Day: Sunday), while the LE training week consisted of three sessions of two hours (Tuesday, Thursday, and Friday; Rest days: Sunday, Monday, Wednesday, and Saturday). The training schedules were planned exclusively by the technical staff without interference from the researchers and started with warm-ups and body exercises.

In terms of load, intensity control was based on the maximal target zone limit considering repetition volume. Increasing peripheral levels, greater specificity and demands, as well as exercise combinations were considered adequate intensity. These adjustments depended on training periodization; generally, the initial or basal period was characterized by high volume demands while specific or pre-competitive periods were based on specific AG exercises (Table 1).

### 2.4. Sports Injuries Incidence

The sports injuries incidence was reported through a morbidity survey [21,22]. This study considers an injury case as tissue damage—or other derangement of normal physical function—due to participation in sports resulting from rapid or repetitive transfer of kinetic energy [23] that limited the exercise training attendance for at least one day, regardless of the need for medical care [21,22]. The total number of injuries was divided by the total exposure time to the training and then multiplied by a factor of 1000 to determine the injury incidence per 1000 h of practice. In general, incidence-based measures that provide a standard time window for the population at risk (injuries per hour) are preferable to measures for which the time at risk varies across individuals (injuries per athletic exposure) [23].

### 2.5. Training Load Monitoring

The duration and frequency of the training and competition sessions were registered. The internal training load (ITL) was determined based on the session rating of the perceived exertion method [24]. The ITL was calculated as the product of the duration of each training session (min) and session rating of perceived exertion score, and reported in arbitrary units (AUs), described according to the total weekly ITL [3,23]. Monotony (MT) was calculated as a training variability index that can be defined from the daily mean/standard deviation values obtaizzned over some time, while strain (ST) was obtained from the product of training load and training monotony [25]. Both variables—MT and ST—offer information on negative adaptations to training [25].

The acute: chronic workload ratio (ACWR) was calculated from weekly ITL values and describes the acute (1-week) workload concerning the chronic (4-week rolling average) workload [2,20]. The ACWR was calculated using coupled acute and chronic workload data [2,25]. A spike, or rapid increase, in training load was defined as an ACWR > 1.5. On days off, the training load was considered zero, a value that was included in the general analysis.

### 2.6. Recovery Monitoring

The TQR scale was used to monitor recovery, according to Kenttä and Hassmén [26]. Before the first session of each day, athletes answered the question, “How do you feel about your recovery?’’, by pointing to a value on a scale from 6 to 20. The weekly average TQR score was calculated based on the daily TQR values from a week analyzed, excluding days off. A score of >13 (reasonable recovery) indicated a minimally adequate recovery state [26].

### 2.7. Statistical Analysis

Results were manually entered and analyzed on the Sigma Stat (Version 3.5; Chicago, IL, USA), and Jamovi^®^ (Version 2.3.28.0) softwares. Considering continuous quantitative variables, the Shapiro-Wilk test was performed to test the normality, and the Levene median test was conducted to analyze equal variance assumptions. Upon not confirming at least one condition, the results were converted into a logarithm scale (Ln) to ensure normal data distribution and/or equal variances. If these conditions persisted, non-parametric proceedings were adopted to analyze the results. The results were presented as descriptive statistical measures. Comparisons between groups regarding general characteristics were performed using the Student t-Test. Data on the participants’ age, body adiposity, and week training exposure were analyzed by the Mann–Whitney Rank Sum test. Load monitoring and recovery variables were evaluated by a Two-Way Repeated-Measures Analysis of Variance (Two-Way RM ANOVA), complemented by Bonferroni’s multiple comparison test. Partial eta squared (η^2^p) was calculated to determine the effect size, defined as follows: Small (>0.0099 and <0.0588), moderate (>0.0588 and <0.1379), and large effects (>0.1379), respectively [27].

An analysis of covariance (ANCOVA) technique was conducted to analyze if load monitoring variables affected recovery results. Categorical variables were presented as absolute and relative (%) proportions and analyzed by Chi-square. The significance level was set at *p* < 0.05 for all statistical analyses.

## 3. Results

**Figure 1:**
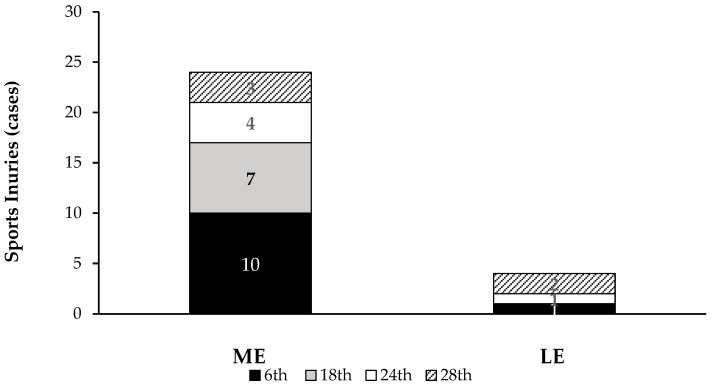
Sports injuries (cases) in gymnasts in accordance with gymnastic performance levels and exercise training periods; ME, medium exposure group; LE, low exposure group; 6th, specific preparatory period; 18th, precompetitive preparatory period; 24th, competitive period; 28th, transition period.

Table 2 shows the general characteristics according to the group. Age values and anthropometric results were similar between the groups—ME and LE (*p* > 0.05). ME presented higher male gymnasts’ proportion and exercise training week practice than LE (*p* < 0.001). In terms of musculoskeletal injuries, a total of 28 cases were registered for 28 weeks. ME participants reported a higher prevalence and incidence of injuries per gymnast, and per 1000 h of practice than LE (Table 2). Female participants from the ME group reported 15 injury cases (62.5%), with 75% prevalence and 1.88 cases/gymnast incidence. In general, during the observed period, 81.3% (n = 13) related injuries in ME, 18.1% (n = 2) confirmed injury incidence in the LE group. In addition, most cases’ onset followed the first exercise training period (Figure 1).

Table 3 shows the training load monitoring variables according to the group and exercise training period. In general, ITL and strain results were about twice as high in ME as in LE at all time points evaluated, sustaining a group’s statistically significant effect. In terms of the training period, ME presented greater ITL from the pre-competitive until the transition period compared to the initial moment (1st–6th week). The strain values were generally increased from specific until the competitive period in both groups (~2 times). Considering monotony, ME demonstrated significantly increased values than the LE group in the first exercise training moment; meanwhile, monotony was significantly reduced in the ME during the last moment. Additionally, LE revealed significantly increased monotony during the specific period (*p* < 0.001). In conclusion, ME exhibited lower ACWR values (~10–15%) than LE in most training periods (Table 3).

Importantly, a notable difference was observed in the ITL outcomes between the analyzed groups (η^2^p = 0.921; *p* < 0.001), as well as across different moments within the training period (η^2^p = 0.253; *p* < 0.001). The monotony variable showed significant differences for the time factor (η^2^p = 0.228; *p* < 0.001) and for the interaction between time and group factors (η^2^p = 0.240; *p* < 0.001). Significant differences in strain were found for time (η^2^p = 0.343; *p* < 0.001), group (η^2^p = 0.540; *p* < 0.001), and their interaction (η^2^p = 0.276; *p* < 0.001). A significant difference was observed between the groups regarding the ACWR (η^2^p = 0.561; *p* < 0.001). All intergroup comparisons for these outcomes exhibited large effect sizes.

**Figure 2:**
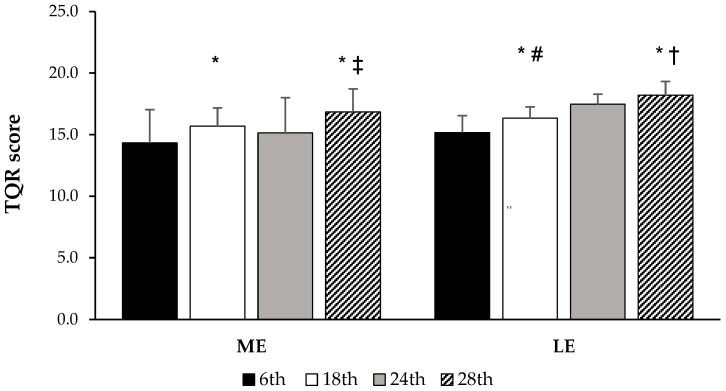
Total quality recovery (TQR) score in gymnasts in accordance with gymnastic exposure levels and exercise training periods; ME, medium exposure group; LE, low exposure group; 6th, specific preparatory period; 18th, precompetitive preparatory period; 24th, competitive period; 28th, transition period. Effects of moment: * *p* < 0.05 vs. 1st–6th week; † *p* < 0.05 vs. 7th–18th week; ‡ *p* < 0.05 vs. 19th–24th week; Effects of group: # *p* < 0.05 vs. ME group; Two-Way RM ANOVA, and Bonferroni’s test.

Figure 2 illustrates the TQR scores according to group and moment. Within the ME group, precompetitive and transition periods were accompanied by significantly increased TQR values compared to the initial moment; likewise, the transition revealed higher TQR than the competitive period. In the LE group, scores significantly increased during competitive and transition periods compared to the specific period, and the transition was also greater than the competitive period.

Considering intergroup comparisons, ME showed significantly reduced TQR scores than LE during the competitive training period (24th week results). Since ITL, Strain, and ACWR were also different between groups at this moment (Table 3), TQR results were adjusted for those variables and reanalyzed (ANCOVA). Upon being adopted as a covariate, strain values were statistically significant, confirming a potential relationship with TQR group differences during the competitive period (Table 4).

## 4. Discussion

This study aimed to analyze internal training load, total quality recovery, and acute: chronic workload ratio, as well as sports injury incidence in gymnasts with different levels of exposure during a sequential sports season. Succinctly, gymnasts subjected to moderate exposure level exhibited greater incidence of musculoskeletal injuries, as well as increased training load values, and impaired recovery during a competitive period within a 28-week follow-up, when compared to gymnasts at a lower exposure level.

Methodologically, these issues were settled by a two-independent group design. In this aspect, similar anthropometric values between groups could be attributed to the comparable age range and competition categories included in the study. Gymnastics athletes often exhibit standardized anthropometric traits suited to their sport [28]. Generally, artistic gymnasts demonstrate higher muscle mass and lower body fat percentages. Kenney, Wilmore, and Costill [29] suggest that 8–16% body fat is optimal for performance in gymnastics. Based on the results, ME and LE groups presented values of 13.3 ± 5.9% and 16.1 ± 8.7%, respectively. Regarding BMI, the findings of previously published studies are consistent with our results. For instance, Sterkowicz-Przybycień et al. [28] report BMI values of 14.7–24.5 kg/m^2^ for male gymnasts in this age group, aligning with the findings of 20.5 ± 2.5 (boys) and 18.5 ± 2.2 (girls) in Pons et al. [30], as well as our findings.

Likewise, athletes at higher exposure and competitive levels tend to report greater perceived training loads [1]. This higher exposure is associated with elevated ITL values at all points in the ME group. More strenuous training routines are associated with higher rates of musculoskeletal injuries in sports [2,10]. Weekly exposure of 17.30 ± 1.74 h in ME athletes, coupled with increased workload, likely explains the higher incidence of injuries in this group (3.09 vs. 2.53 injuries/1000 h). In this context, combining training intensity and duration, as well as daily RPE assessments, allowed us to calculate exercise training volume [24]. Mesocycles with the highest ITL values occurred during pre-competition and competition phases (moments 2 and 3), consistent with findings from other studies that highlight the intense, repetitive nature of training during these periods aimed at maximizing competitive performance [4,26]. Moreover, when considering only female participants, more accentuated differences between ME and LE groups were found in terms of sports injury incidence. Indeed, the injury risk may be directly associated with the competitive level; i.e., a higher exposure to musculoskeletal demands in training and/or competitions could to lead to a greater probability of injury onset [31]. Intriguingly, elite and sub-elite gymnasts exhibited 2.63 and 4.11 injuries per 1000 h, respectively, for an 18-month prospective period [32]. Gymnasts competing at the sub-elite level, and with inadequate technical improvement when compared to elite gymnasts, may be subjected to a greater risk of injury. On the other hand, elite gymnasts reported significantly higher injury rates per year than the sub-elite participants [23].

In terms of monotony, Foster et al. [33] indicated that values exceeding 2.0 could be associated with overtraining, indicating potential performance declines and increased injury risk. Indeed, most evaluated periods showed monotony values > 2.0 AU, reflecting limited variability in terms of training loads without sufficient recovery periods. It is noteworthy that transition and specific periods constituted exceptions in ME and LE, respectively. In contrast, high monotony does not always result in overtraining, as 52% of elevated monotony peaks are not associated with any reported illness [25]. Strain, defined as the product of training load and monotony, revealed similar patterns, increasing with higher weekly loads or monotony values. Strain values exceeding 6000 AU may indicate overtraining, although 59% of deviations in strain are not linked to illness [25]. It is worth noting that our results did not reach this magnitude (6000 AU).

Importantly, acute and chronic training loads vary significantly across sports [2]. Acute loads can represent a single day of exposure, while chronic loads typically are considered from an average of the last 3–6 weeks; in addition, a 4-week moving average was used as a parameter in this investigation. Acute loads represent fatigue, while chronic loads indicate “fitness” [34]. For balanced athlete training, acute and chronic loads should align closely, with an ACWR near 1.0 indicating optimal conditioning [3]. Based on this, we found significant differences between groups (*p* < 0.001), with LE athletes consistently showing ACWR values above 1.0, remaining within the “protection zone” against injuries. Gabbett et al. [35] noted that ACWR values between 0.8 and 1.3 are ideal for minimizing injury risk. Malone et al. [36] found values between >1.00 and <1.25 to be protective compared to groups with ACWR ≤ 0.8. In this perspective, ME athletes showed ACWR values between 0.952 and 0.972, which could be associated with an increased likelihood of injury onset when considering the LE values (>1.00). In contrast, ACWR > 1.5, where acute load far exceeds chronic load, increases injury risk [6], which could be observed in athletes with week training exposure above 20 h [19].

In parallel, the TQR scale reflects athletes perceived recovery after training. It is suggested that recovery values should not drop below 13 points [26], even after consecutive days of light training. Both ME and LE groups maintained TQR scores within a safe range, consistently above 13 throughout the study. Notably, TQR scores increased during moment 4, when training intensity decreased, coinciding with a reduction in injury incidence. During the competition phase (competitive exercise training), ME athletes reported significantly lower TQR scores than LE participants, likely reflecting increased fatigue levels in the ME group. Indeed, peak strain values were observed during the competitive period in ME, and analysis of covariance highlighted the role of this variable by negatively impacting the recovery status during this period (Table 4). As the incidence of injuries was not impacted by these circumstances, other risk factors may configure potential backgrounds for injury onset, in addition to exercise training demands. From this pathophysiological perspective, a complete understanding of the potential causes of sports injuries must address the multifactorial nature of musculoskeletal injuries in any sports modality, integrating not only extrinsic but also intrinsic risk factors [2,34,36]. Therefore, it cannot be ruled out that internal load alterations are part of this etiological combination.

In terms of clinical relevance, it is worth mentioning that internal training load, as well as recovery conditions, influenced injury onset in ME gymnasts, particularly during a competitive period. In this context, the analysis of those variables could be useful as a complementary method for monitoring sports injuries during the training and competition seasons. Within this perspective, physiological variables, biomechanical factors, and anthropometric characteristics may be linked to the internal load demands and musculoskeletal injuries onset. Further studies should address these parameters to better investigate adaptive alterations in response to sports season artistic gymnastic training.

Regarding other limitations, our results on internal load were based on subjective effort perception, which can be influenced by individual factors of each athlete, as well as its understanding, given that the investigation incorporated mostly young and sub-elite participants. It is expected that different and more direct associations among internal load, recovery, and sports injury incidence are observed in gymnasts subjected to weekly training volume > 20 h [19].

## 5. Conclusions

In conclusion, increased training load values showed to be linked to the incidence of musculoskeletal injuries in gymnasts subjected to different training week exposure levels. Likewise, the high values of internal training load were related to impaired recovery during a competitive period within a 28-week follow-up.

## Figures and Tables

**Table 1:** Description of exercise training periods of artistic gymnasts.

Period Weeks	Weeks	Characteristics
Specific Preparatory	1–6	General conditioning, mainly flexibility, aerobic capacity, and strength; promotion of new morphologic adaptations in the athlete’s body after vacation and the composition of new routines
Precompetitive Preparatory	7–18	Decrease in duration of general conditioning and greater duration and intensity of specific conditioning; increase in technical training duration and intensity. Improvement of competitive performance, increase in the specificity in all components of the training session
Competitive	19–24	Peak performance during the competition of the season; focus on technique and routine, with high intensity and repetition; adjustment of training
Transition	25–28	Exercise training new elements

**Table 2:** General features.

Variable		Group		*p*-Value
		ME	LE	
Age (years)		10.0 (8.5; 11.0)	11.0 (9.3; 11.0)	0.252
Heigth (cm)		138.1 ± 9.1	144.7 ± 13.1	0.132
Body Weight (kg)		34.6 ± 7.8	39.1 ± 10.5	0.211
BMI (kg/m^2^)		17.9 ± 2.1	18.4 ± 2.9	0.578
Body adiposity (%) ^a^		11.8 (8.6; 14.7)	12.7 (9.7; 18.7)	0.361
Practice Time (years)		4.2 ± 1.5	3.6 ± 2.0	0.412
Week training exposure (h) ^a^		17.38 (16.37; 18.79)	5.14 (5.00; 5.21)	<0.001 *
Gender	Female	8 (50%)	11 (100%)	0.018 †
	Male	8 (50%)	0 (0%)	
Injuries	Cases incidence	24	4	-
	Cases/gymnasts	1.50	0.36	-
	Cases/injured gymnasts	1.85	2.00	-
	Injuries/1000 h	3.09	2.53	-
Participants (n)		16	11	-

Values expressed in mean ± standard deviation; Student’s *t*-test (*p* > 0.05). ^a^ values expressed in median and interval between 25th and 75th percentiles; * *p* < 0.05, Mann–Whitney Rank Sum Test. Gender expressed in absolute and relative (%) proportions; † *p* < 0.05, Chi-square test. Injuries and participants were expressed as absolute measures.

**Table 3:** Load monitoring variables according to group and exercise training moment.

Variable	Gr	Exercise Training Period (week, W)			
		Specific (1st–6th W)	Precompetitive (7th–18th W)	Competitive (19th–24th W)	Transition (25th–28th W)
ITL	ME	1292 ± 237	1502 ± 215 *	1424 ± 107 *	1449 ± 165 *
	LE	651 ± 63 #	783 ± 110 *#	676 ± 75 #†	750 ± 53 *#
Monotony	ME	4.83 ± 1.98	4.72 ± 1.98	5.74 ± 2.49	2.29 ± 2.43 *†‡
	LE	2.94 ± 0.80 #	5.99 ± 1.56 *	4.53 ± 1.53	4.52 ± 1.6 #
Strain ^a^	ME	3822 (3201)	5951 (2860)	7593 (2800) *	1470 (4242) †‡
	LE	1035 (438) #	2201 (999) *#	1736 (1746) #	2191 (1086) *#
ACWR	ME	0.952 ± 0.126	0.996 ± 0.056	0.972 ± 0.047	1.002 ± 0.031
	LE	1.065 ± 0.135 #	1.018 ± 0.046	1.073 ± 0.073 #	1.118 ± 0.077 #†

Values expressed in mean ± standard deviation; ^a^ Strain values expressed in median and interval between 25th and 75th percentiles. Gr, group; ME, medium exposure group; LE, low exposure group; ITL, internal training load; ACWR, acute: chronic workload ratio; Effects of training period: * *p* < 0.05 vs. Specific; † *p* < 0.05 vs. Precompetitive; ‡ *p* < 0.05 vs. Competitive; Effects of group: # *p* < 0.05 vs. ME group; Two-Way RM ANOVA, and Bonferroni’s test.

**Table 4:** Measurements of analysis of covariance (ANCOVA) for total quality of recovery—TQR results (Group), and the respective covariate during the competitive period (24th week of evaluation).

Variable	Sum of Squares	DF	Mean Square	F	*p*-Value
Group	25.779	1	25.779	6.8656	0.016 *
ITL	0.201	1	0.201	0.0534	0.819
Monotony	16.008	1	16.008	4.2634	0.052
Strain	29.523	1	29.523	7.8627	0.011 *
ACWR	1.329	1	1.329	0.3540	0.558
Residual	78.852	21	3.755		

ITL, internal training load; ACWR, acute: chronic workload ratio; * *p* < 0.05; Analysis of Covariance (ANCOVA).

## Data Availability

The data presented in this study are available on request from the corresponding author.
